# A nurse-led coaching intervention with home telemonitoring for patients with heart failure: Protocol for a feasibility randomized clinical trial

**DOI:** 10.1016/j.mex.2024.102832

**Published:** 2024-06-30

**Authors:** Ines Basso, Erika Bassi, Silvia Caristia, Angela Durante, Cristian Vairo, Salvatore Giuseppe Rocco Patti, Mario Pirisi, Mauro Campanini, Marco Invernizzi, Mattia Bellan, Alberto Dal Molin, INTERCOACH Group, Francesca Caldera, Domenico D'amario, Grabriele Dell'Era, Inelsy Gomez, Alessandra Lazzati, Elena Massara, Claudia Milanese, Antonella Molon, Marta Petteneo, Salvatore Scaramuzzino, Cristina Torgano, Patrizia Zumbo

**Affiliations:** e University of Piemonte Orientale Amedeo Avogadro, Novara, Italy; f University Hospital Maggiore della Carità, Novara, Italy; a University of Piemonte Orientale Amedeo Avogadro, Novara, Italy; b Sant'Anna School of Advanced Studies, Health Science Interdisciplinary Center, Pisa, Italy; c Fondazione Toscana “Gabriele Monasterio”, Pisa, Italy; d University Hospital Maggiore della Carità, Novara, Italy

**Keywords:** Heart failure, Mentoring, Telemonitoring, Transitional care, Hospitalizations

## Abstract

Poor treatment adherence and lack of self-care behaviors are significant contributors to hospital readmissions of people with heart failure (HF). A transitional program with non-invasive telemonitoring may help sustain patients and their caregivers to timely recognize signs and symptoms of exacerbation.

We will conduct a Randomized Clinical Trial (RCT) to evaluate the feasibility and acceptability of a 6-month supportive intervention for patients discharged home after cardiac decompensation. Forty-five people aged 65 years and over will be randomized to either receive a supportive intervention in addition to standard care, which combines nurse-led telephone coaching and a home-based self-monitoring vital signs program, or standard care alone. Four aspects of the feasibility will be assessed using a mixed-methods approach: process outcomes (e.g., recruitment rate), resources required (e.g., adherence to the intervention), management data (e.g., completeness of data collection), and scientific value (e.g. 90- and 180-day all-cause and HF-related readmissions, self-care capacity, quality of life, psychological well-being, mortality, etc.). Participants will be interviewed to explore preferences and satisfaction with the intervention. The study is expected to provide valuable insight into the design of a definitive RCT.

## Graphical abstract

**Figure:**
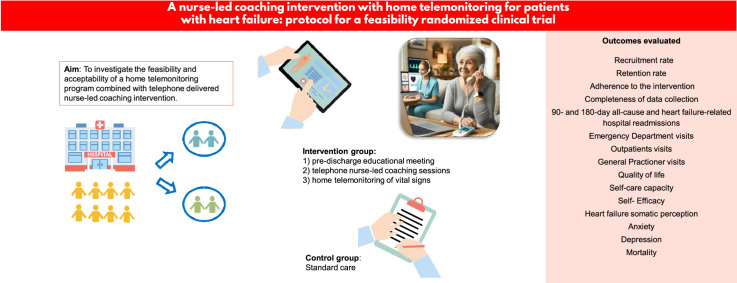

Specifications table

**Table:** 

Subject area:	Medicine and Dentistry
More specific subject area:	*Geriatric care*
Name of your protocol:	INTERCOACH
Reagents/tools:	*Mini-COG* *Edmonton Symptom Assessment System (ESAS)* *Cumulative Illness Rating Scale (CIRS)* *European Health Literacy Survey* *SF-12 Questionnaire* *Self-Care Of Heart Failure (SCHF v. 7.2)* *Heart Failure Somatic Perception Scale v.3 (HFSPS)* *Self-Efficacy Scale* *Geriatric Depression Scale short version* *Hamilton Anxiety Scale*
Experimental design:	*A prospective, open label randomized controlled feasibility study will be conducted, with a 1:2 ratio between intervention and control groups. Participants assigned to the intervention group will receive the following educational program in addition to standard care: 1) an in-person educational intervention before discharge, following the recommendations of the European Society of Cardiology; 2) a nurse-led telephone coaching service (once a week for the initial month, followed by sessions every two weeks); 3) a daily home-based self-telemonitoring system for weight, blood pressure, heart rate, and oxygen saturation. Vital signs will be transmitted to a platform and remotely monitored by a nurse. A structured intervention protocol will be activated based on the patient's clinical conditions.*
Trial registration:	*The study protocol has been registered in the Clinicaltrials.gov:**NCT06285565*
Ethics:	*The study protocol was submitted and approved by the Ethics Committee of Maggiore della Carità Hospital of Novara in December 2023 (*CE390/2023). *The study will adhere to the Ethical Principles of the Declaration of Helsinki, as well as the European Union Standards of Good Clinical Practice* *The data from the feasibility study will be combined with those from the definitive study if it validates the suggested intervention and no substantial corrections will be required.*
Value of the Protocol:	(1) *This protocol is important to inform the design of a definitive RCT, aimed to assess the effectiveness of a remote nurse-led coaching intervention with a home telemonitoring program.* (2) *Given the complexity of the supportive intervention, it is crucial to involve patients in the research process and to collect their preferences regarding the intervention.* (3) *3) This feasibility study is important to define a successful care plan that guarantees follow-up for patients with heart failure*.

## Background

The protocol aims to develop and evaluate a complex healthcare intervention intended to enhance the care of patients discharged home following a cardiac decompensation. Transitioning from hospital to home poses significant challenges for older adults with chronic diseases, making a supportive educational intervention crucial to facilitate this process and prevent complications [[1], [2], [3]].

Generally, transitional care programs offer time-limited services and person-centered interventions to ensure continuity of care and avoid adverse events [4]. Over the past two decades, several evidence-based models have been developed [1,5]. These models include various interventions such as home visits, multidisciplinary teams, patient and caregiver self-care education, and medication reconciliation [6]. Although beneficial, these standard in-person interventions are expensive and hardly sustainable given the progressive aging of the population and the increasing incidence of chronic diseases [7,8]. Information and communication technologies enable innovative approaches to deliver care remotely, potentially reducing costs and increasing the scalability and accessibility of these interventions.

The proposed intervention is designed to tele-monitor and support the patient's self-care capacity from a distance. Given its complexity, which arises from the involvement of various stakeholders, the transition between diverse settings, and the presence of multifaceted program elements, conducting a feasibility study becomes a critical step in designing a definitive trial. Testing in advanced the intervention enables investigators to explore uncertainties and refine implementation, ultimately enhancing its effectiveness [9].

## Description of protocol

Heart failure represents a significant global health challenge, with an increasing prevalence of 0.6 % annually [10]. Home management of the disease can be daunting, especially following a hospitalization, when patients may encounter difficulties in adjusting their lifestyle to comply with the recommendations provided [11,12]. Indeed, poor treatment adherence and lack of self-care behaviors are important factors contributing to hospital readmissions [13,14].

A telephone-based supportive intervention led by nurses has the potential to enhance the self-care capacity of patients and their caregivers, thereby promoting the maintenance of healthy lifestyles. Similarly, non-invasive home telemonitoring could serve as a beneficial strategy for preventing acute decompensation by allowing healthcare professionals to remotely monitor individuals' vital signs and detect early signs of exacerbation.

Nevertheless, the effectiveness of this approach remains inconclusive even due to heterogeneity across studies, including differences in patient acuity, the level of educational support provided, follow-up durations, and the technology utilized. [[15], [16], [17], [18], [19]].

### Aim of the study

The primary aim of the study is to investigate the feasibility and acceptability of a home telemonitoring program combined with telephone-delivered nurse-led coaching intervention.

The secondary aim is to explore the feasibility of a randomized controlled study to assess the effectiveness of the supportive intervention.

## Methods

### Study design and randomization

A parallel, open label randomized controlled feasibility study will be carried out utilizing a mixed method approach. The protocol complies with the criteria reported by SPIRIT 2013 statement [20]. We will generate a list of randomizations stratified by wards and gender, with a 1:2 ratio between intervention and control groups, using web-based software.

### Population and enrollment

Patients will be enrolled in three medical wards of a large University Italian hospital at the time of discharge and will be followed in the domiciliary setting. Sequentially numbered sealed, opaque envelopes will be used to conceal the allocation.

### Inclusion and exclusion criteria

All patients aged 65 and over, hospitalized for cardiac decompensation, regardless of ejection fraction value (preserved or decreased), and expected to be discharged home will be considered eligible.

Individuals who lack the cognitive and/or physical capabilities (POSITIVE Mini-COG)) [21] for self-monitoring of vital signs, and without a caregiver available to assist them, will be excluded. In addition, individuals who are receiving ongoing medical care from other services will also be excluded.

### Sample size

As this is a feasibility study, it is not required to calculate the sample size [22]. Instead, the sample must be representative of the reference population [22]. Accordingly, it is planned to recruit 45 patients (15 will be allocated to the intervention arm and 30 to the control arm).

### Intervention group

A supportive program will be provided in addition to standard care, consisting of 1) a pre-discharge educational meeting, 2) telephone nurse-led coaching sessions and 3) home telemonitoring of vital signs.

(1) *Pre-discharge educational meeting.* After the patient is deemed stable by the clinicians, an in-hospital educational intervention will be provided by a trained nurse not involved in the clinical pathway. Family members will be invited to participate but attendance will not be mandatory. Key topics of self-care management recognized by the European Society of Cardiology will be discussed, including the importance of physical activity, rest and sleep quality, fluid restriction, a healthy diet, medication adherence, the advantages of immunization, smoking cessation, psychological issues, need for formal and informal support [23].
  During the meeting, the nurse will use the Transtheoretical Model of Prochaska & DiClemente [24] to identify the individual's priority goals and motivation for change, thus initiating the coaching session. The model recognizes five stages of change:
  - pre-contemplation: not yet recognizing the existence of problematic behavior that requires change.
  - contemplation: recognizing the presence of a problem but not yet being prepared, certain, or confident about the desire or ability to enact a change.
  - determination: preparing to change behavior.
  - action: taking action to change behavior.
  - maintenance: maintaining the changed behavior.
(2) *Telephone nurse-led coaching sessions.* In this phase, patients will be encouraged to focus on their values and progress towards their goals. The intervention will be tailored based on the stage for change and personal difficulties reported by the individual during the initial meeting (e.g. if the patient is in the pre-contemplation phase, the nurse will adopt an informative approach). The scheduled sessions will occur weekly during the first month and then transition to twice a month thereafter. If the person is unavailable, a second attempt will be made the following day at the same time.
(3) *Home telemonitoring of vital signs*. Patients will receive education on measuring weight, blood pressure, heart rate, and oxygen saturation at rest every morning before breakfast. All participants will be provided with a telemonitoring system connected to a smartphone, which acts as a gateway and transmits data to a web platform via Bluetooth.

The system will be equipped with alarms to signal deviations in vital signs from predefined ranges. Upon notification, the nurse will contact the patient within 72 h (excluding Saturdays, Sundays, and holidays) to assess the presence of symptoms and/or signs of congestion. Patients will be asked the following questions: “Are you short of breath?” Describe the sensation on a 0–10 scale, where 0 means no shortness of breath and 10 severe shortness of breath. By the Edmonton Symptom Assessment System (ESAS) scale [25], scores ≤4 will be considered mild; 5–7 moderate; ≥ 8 severe. “By applying pressure with your finger to the area near the ankle, do you observe an indentation? Have you had trouble tying your shoes in the last few days?” The questions will be assessed on dichotomous (yes/no) scales. Depending on the conditions of the person, a multidisciplinary protocol will be implemented (Table 1).

**Table 1:** Intervention protocol in the event the nurse identifies an alteration in vital signs through remote telemonitoring.

Signs and symptoms alterations	Multidisciplinary interventions
*Systolic blood pressure* <90 or >150 mm/Hg and/or *Heart rate* <60 or >100 bpm and/or *± 2kg in the last three days.* *and/or* *≤92 % oxygen saturation* **Without or mild symptoms** (ESAS *Score Scale ≤4)*	Self-management interventions will be reinforced as needed, with re-evaluation in the days following. If alterations persist for three consecutive days, referral to the patient's physician will be recommended
*Systolic blood pressure* <90 or >150 mm/Hg and/or *Heart rate* <60 or >100 bpm and/or *± 2kg in the last three days.* *and/or* *≤92 % oxygen saturation* **With moderate symptoms** (ESAS Score Scale 5–7)	The nurse will request an assessment or consultation from the physician of the hospital ward where the patient was admitted
*Systolic blood pressure* *<90 or >150mm/Hg* *and/or* *Heart rate* *<60 or >100 bpm* *and/or* *± 2kg in the last three days* *and/or* *≤92 % oxygen saturation* **With severe symptoms** (ESAS Score Scale ≥ 8)	Referral to the emergency service

Before hospital discharge, the physician will specify a dry weight to be maintained and the dosage of a loop-type diuretic (i.e., torsemide or furosemide) for clinical stability. In the event of a weight gain exceeding 2 kg within three days (with no other symptoms), consistent with previous days and under identical conditions, the diuretic dosage will be doubled for three days [23].

The patients can contact the nurse by telephone from Monday through Friday from 8:30 a.m. to 5:30 p.m. or request a home visit. This service does not replace the emergency services.

### Control group

Participants who will be randomized to the control group will receive the usual care, which follows clinical guidelines [23].

For patients admitted to one out of three wards, an outpatient visit will be scheduled one month after discharge. However, no follow-up will be planned for patients discharged from the other two wards.

### Outcomes measured

Four aspects of feasibility will be the focus of the outcomes’ measurement [22]:

- Process: evaluates the feasibility of the study's fundamental steps (e.g. recruitment rate).
- Resource: investigates potential time and resource problems during the study (e.g. retention rate, adherence to the coaching intervention, adherence to the telemonitoring program, and the amount of time spent by nurses each week on coaching interventions).
- Management: examines possible issues related to patient and data handling (e.g. frequency of calls made by patients to the nurse, the acceptability of the intervention to patients, completeness of data collection).
- Scientific value: describes how treatment safety, response, effect, and effect variance are evaluated (patient's self-care capacity, self-efficacy, quality of life, somatic perception of the disease, emotional and psychological well-being, mortality, all-cause hospital admissions, HF-related hospital admissions, etc.)

## Data collection

To ascertain whether the intervention is acceptable and feasible, both quantitative and qualitative data will be obtained. Quantitative data will be collected via telephone by an assessor not involved in the coaching intervention and blinded to the group assignment.

### Quantitative data

Table 2 outlines the types and timeframes of the variables measured.

**Table 2:** Variables and timeframes for measurements.

	T0 (baseline)	T1 (3 months)	T2 (4 months)	T3 (6 months)
Recruitment rate				**×**
Retention rate				**×**
Adherence to the coaching intervention			**×**	
Adherence to telemonitoring program			**×**	
Weekly time dedicated to coaching intervention but the nurse			**×**	
Number of returned questionnaires	**×**	**×**		**×**
Semi-structured individual interviews			**×**	

Sociodemographic information	**×**			
Comorbidities	**×**			
Number of medications	**×**	**×**		**×**
Left ventricular ejection fraction	**×**			
New York Heart Association (NYHA) Functional Classification	**×**		**×**	
B-type natriuretic peptide (BNP) dosing	**×**			
Number of hospital admissions in the past two years	**×**			
Anthropometrical indicators	**×**	**×**		
Health literacy	**×**			
Cognitive status	**×**	**×**		**×**
Vaccinations	**×**	**×**		**×**
Smoking habits	**×**	**×**		**×**
Heart failure-related hospital readmissions		**×**		**×**
All-cause hospital readmissions		**×**		**×**
Outpatient visits		**×**		**×**
General Practitioner visits		**×**		**×**
Emergency Department visits		**×**		**×**
Quality of life	**×**	**×**		**×**
Self-care capabilities	**×**	**×**		**×**
Self-efficacy	**×**	**×**		**×**
Heart failure somatic perception	**×**	**×**		**×**
Anxiety	**×**	**×**		**×**
Depression	**×**	**×**		**×**
Mortality		**×**		**×**

Sociodemographic and clinical data will be collected:

*Sociodemographic characteristics:* gender, age, education level, marital status, social network, employment status, financial level.

*Clinical information*: comorbidities according to the Cumulative Illness Rating Scale (CIRS) [26], anthropometrical indicators*,* left ventricular ejection fraction (considered reduced for values 〈40 %; mildly reduced between 41 %- and 49 %; preserved if 〉 50 %), B-type natriuretic peptide (BNP) dosage; the number of medications, number of hospital admissions in the past two years, staging of HF according to New York Heart Association (NYHA) Functional Classification, level of health literacy according to the European Health Literacy Survey (short-short form, English version) (HLS-EU-Q6) [27], Mini-COG [21] smoking habit, weight and height, COVID-19 and pneumococcal vaccinations.

The following outcomes will be measured:

- *Recruitment rate:* the proportion of patients who agreed to participate relative to those who fulfilled the inclusion criteria.
- *Retention rate*: the proportion of patients who complete the study relative to those who consent to participate.
- -*Adherence to the coaching intervention:* the number of coaching phone calls scheduled relative to those actualized.
- *Adherence to telemonitoring program:* the proportion of days during which vital signs were measured and sent by the system relative to the total duration of the intervention.
- *Weekly time dedicated to coaching intervention by the nurse* (in hours).
- *Completeness of data collection:* the number of returned questionnaires.
- *Number of weekly calls made by patients to the nurse.*
- *Quality of life*: Quality of life is a value that integrates objective (physical health, personal circumstances, social relationships, social and economic influences) and subjective indicators (such as how the individual responds to objective conditions), related to various dimensions of life and personal values [28]. This outcome will be assessed using the Italian version of the SF-12 scale [29]. This is a multidimensional scale consisting of 12 questions related to physical functioning, limitations due to physical health or emotional factors, energy and fatigue, emotional well-being, social activities, pain, and general health perception. The scale collects self-report data using a Likert scale. The score for each subscale will be standardized on a 0–100 scale, where higher values indicate better quality of life.
- *Self-care capacity*: The self-care capacity of the patient encompasses three dimensions: self-care maintenance, symptom perception, and self-care management [30]. Self-care maintenance reflects the active commitment and responsibility for one's own care, as well as the behaviors adopted to maintain physical and emotional stability (e.g., adherence to therapy). On the other hand, symptom perception refers to the ability to respond to signs and symptoms of disease exacerbation when they occur, such as by searching for help (self-care management) [30]. The self-care capacity will be evaluated using the Italian version of the Self-Care of Heart Failure Index (SCHF v. 7.2) [31]. The tool consists of three different scales: self-care maintenance (10 items), symptom perception (11 items), and self-care management (8 items) producing a standardized score of 0–100, where higher values refer to the greater self-care capacity. A score ≥70 indicates adequate self-care capacity.
- *Self-Efficacy Scale:* Self-efficacy perception refers to the individual's belief in their ability to achieve certain goals because of their actions, regardless of the challenges and difficulties they may face [32]. Self-efficacy is a mediator of self-care capacity [32]. A scale consisting of 10 items using a 5-response Likert scale will be utilized [33].
- *Heart Failure Somatic Perception Scale v.3* (HFSPS) [34]. HF is a complex clinical syndrome characterized by several signs and symptoms, including shortness of breath, fatigue, and peripheral fluid accumulation [23]. To measure this outcome, we will employ a scale comprising four factors, which evaluate the presence and impact of 18 typical signs and symptoms of HF experienced by the patient in the preceding week. Each item has 6 response options ranging from 0 (I do not have this symptom) to 5 (extremely severe), with a range from 0 to 90, where high scores lead back to a greater impact of symptoms on the patient.
- *Geriatric Depression Scale short version* (GDS) [35] for detecting depressive symptoms of the elderly. It consists of 15 questions involving a dichotomous response (yes/no). Ten questions are indicative of depression for positive responses, while the remaining five (items 1, 5, 7, 11, 13) for negative responses. Scores 0–4 are considered normal, depending on age and education; scores between 3 ± 2 are associated with the absence of depressive symptoms, 7 ± 3 mild depression, and 12 ± 2 depression.
- *Hamilton Anxiety Scale* [36]: for the assessment of anxiety symptoms (psychological and somatic). The scale consists of 14 items, rated on a 4-point Likert scale (0=not present; 4= severe), with a range of total score from 0 to 56, where values ≤ 17 indicate mild anxiety, 18–24 moderate anxiety and ≥ 25 severe anxiety.
- *Hospital readmissions at 90- and 180- days* after hospitalization for all causes and HF-related, Emergency Departments visits, outpatient visits, General Practitioner visits, and mortality.

### Qualitative data

To explore perceived barriers and facilitators to the intervention, as well as acceptability and satisfaction with participation, individual semi-structured interviews will be conducted [37]. The interviews will take place after 4 months and will be held at the most convenient location for the participants (home, hospital outpatient clinic or via telematics). To facilitate the participants’ response a track guide will be followed (Table 3).

**Table 3:** Interview guide.

Questions
Q1) Please could you tell me what prompted you to participate in the study?
Q2) Thinking back to the meeting you had with the nurse before discharge, do you remember what you talked about? How did you feel during that meeting? In your opinion, was it helpful/important?
Q3) Once at home, did you have difficulty using the system for measuring vital signs? Did you need someone to help you?
Q4) Regarding self-monitoring, were you able to measure your vitals every day as required?
Q5) Compared to your telephone support intervention up to now, how do you rate the quality of the support you receive? Do you feel that the way of communication used has influenced the accuracy of the information provided?
Q6) Can you remember situations in which you feel you did not receive adequate support? Can you tell me about that incident? In your opinion, what could have been done differently? Why?
Q6) Overall, do you feel satisfied with the care you received? If you had the opportunity, what would you change about the suggested intervention? Why?

Each participant will sign an independent informed consent before beginning the interview. Confidentiality will be guaranteed by using identification numbers. The interview can be interrupted by the patient at any time.

### Statistical methods

Data will be stored using REDCap (Research Electronic Data Capture), a secure web-based application developed to support data capture [38]. For quantitative data, a descriptive analysis will be conducted, considering the nature of the variables. If feasible, comparisons of characteristics and outcomes between the intervention and control groups will be made using parametric or non-parametric tests, depending on the distribution of the variables.

For qualitative data, a qualitative descriptive approach will be used [39]. Interviews will be audio-recorded and transcribed *verbatim* within 48 h. Data will be analyzed according to content analysis method [40], which comprises the following steps. 1) Familiarization: the interviews will be carefully and repeatedly read; 2) Compilation: two independent researchers will review each interview, identifying the most significant words and phrases (units of meaning); 3) Condensation: a descriptive label (code) will be attributed to each meaning unit; 4) Categories: similar codes will be grouped into categories; 5) Themes: similar categories will be condensed into themes.

### Ethics statement

The study protocol was submitted and approved by the Ethics Committee of Maggiore della Carità Hospital of Novara in December 2023 (CE390/2023). The study will adhere to the Ethical Principles of the Declaration of Helsinki, as well as the European Union Standards of Good Clinical Practice.

## Protocol validation

To the best of our knowledge, this protocol has not been implemented in other settings before, therefore preliminary data are not available.

## Limitations

The successful implementation of this protocol hinges on patients' adherence to the educational intervention and tele-monitoring program. Given that heart failure is an age-related condition, participants are likely to be older and potentially less familiar with technological devices. Although training in the use of telemonitoring devices is offered in hospital setting, participants may encounter challenges in accurately measuring their vital signs once they return home. To address this potential issue, provisions have been made for nurses to conduct home visits if the patients will encounter difficulties.

## CRediT authorship contribution statement

**Ines Basso:** Conceptualization, Methodology, Writing – review & editing. **Erika Bassi:** Supervision, Conceptualization, Visualization. **Silvia Caristia:** Software. **Angela Durante:** Visualization. **Cristian Vairo:** Writing – original draft. **Salvatore Giuseppe Rocco Patti:** Supervision. **Mario Pirisi:** Supervision. **Mauro Campanini:** . **Marco Invernizzi:** Project administration, Funding acquisition. **Mattia Bellan:** Project administration, Funding acquisition. **Alberto Dal Molin:** Project administration, Funding acquisition, Supervision, Methodology.

## Declaration of competing interest

The authors declare that they have no known competing financial interests or personal relationships that could have appeared to influence the work reported in this paper.

## Data Availability

No data was used for the research described in the article.
